# circELP2 reverse‐splicing biogenesis and function as a pro‐fibrogenic factor by targeting mitochondrial quality control pathway

**DOI:** 10.1111/jcmm.18098

**Published:** 2023-12-30

**Authors:** Songzi Zhang, Diwei Tu, Weili Liu, Ruiqiong Li, Mengqi Jiang, Xinglong Yuan, Jianlin Luan, Hongbo Li, Changjun Lv, Xiaodong Song

**Affiliations:** ^1^ Department of Respiratory and Critical Care Medicine Binzhou Medical University Hospital, Binzhou Medical University Binzhou China; ^2^ Department of Cellular and Genetic Medicine Binzhou Medical University Yantai China; ^3^ Department of Clinical Nursing Binzhou Medical University Hospital, Binzhou Medical University Binzhou China

**Keywords:** circRNA, miRNA, mitochondrial quality control pathway, pulmonary fibrosis, YAP1/TAZ

## Abstract

Idiopathic pulmonary fibrosis (IPF) is considered as a chronic, fibrosing interstitial pneumonia with unknown mechanism. The present work aimed to explore the function, biogenesis and regulatory mechanism of circELP2 in pulmonary fibrosis and evaluate the value of blocking circELP2‐medicated signal pathway for IPF treatment. The results showed that heterogeneous nuclear ribonucleoprotein L initiated reverse splicing of circELP2 resulting in the increase of circELP2 generation. The biogenetic circELP2 activated the abnormal proliferation and migration of fibroblast and extracellular matrix deposition to promote pulmonary fibrogenesis. Mechanistic studies demonstrated that cytoplasmic circELP2 sponged miR‐630 to increase transcriptional co‐activators Yes‐associated protein 1 (YAP1) and transcriptional co‐activator with PDZ‐binding motif (TAZ). Then, YAP1/TAZ bound to the promoter regions of their target genes, such as mTOR, Raptor and mLST8, which in turn activated or inhibited the genes expression in mitochondrial quality control pathway. Finally, the overexpressed circELP2 and miR‐630 mimic were packaged into adenovirus vector for spraying into the mice lung to evaluate therapeutic effect of blocking circELP2‐miR‐630‐YAP1/TAZ‐mitochondrial quality control pathway in vivo. In conclusion, blocking circELP2‐medicated pathway can alleviate pulmonary fibrosis, and circELP2 may be a potential target to treat lung fibrosis.

## 1 INTRODUCTION

Idiopathic pulmonary fibrosis (IPF) is an insidious and fatal interstitial lung disease accompanied by fibrotic response, including epithelium injury, fibroblast activation, abnormal proliferation and migration and extracellular matrix (ECM) accumulation. The common clinical characteristics are difficulty breathing and dry cough. Lung function tests demonstrate restrictive damage and declined carbon monoxide‐diffusing capacity. Ultimately, patients die of respiratory failure.^1^ However, the mechanism involved in IPF progression is still remain poorly understood. Increasing reports have found that circular RNAs (circRNAs) are critical to the development of lung fibrosis, including alveolar epithelium injury,^2^ myofibroblast proliferation and activation,^3^ macrophage activation,^4^ and abnormal autophagy.^5^ These circRNAs crosstalk with protein‐coding or/and non‐coding genes to promote or inhibit pulmonary fibrogenesis.^6^ So study of mechanism of circRNA as well as therapeutic target can bring new energy to diagnose and treat pulmonary fibrosis.

The circRNA is a covalently closed ring structure produced from a noncanonical RNA back‐splicing event and not easily degraded by exonuclease, hence its greater stability compared with the linear mRNA. Taking advantage of this high‐stability feature, the circRNA^RBD^ vaccine enables higher and more durable antigen production than the mRNA vaccine, making it a favourable choice against SARS‐CoV‐2 variants.^7^ However, functions of circRNAs are still unclear because the accurate technologies that can identify circRNAs from cognate mRNAs with overlapping exons are still lack. Recently, Chen et al. have established CRISPR–RfxCas13d tool to screen regulatory circRNAs at both the individual and large‐scale levels. By screening the circRNAs that take part in cell growth, scholars found that the most of circRNAs not only play generic housekeeping roles but rather function in a regulatory and cell/tissue‐type‐specific manner.^8^ Therefore, following the development of biotechnology, finding and analysing circRNAs has important clinical application value for drug action targets, gene therapy drugs or diagnostic biomarkers.^9^, ^10^, ^11^

In our previous study, the dramatically dysfunctional circRNAs were screened out from the plasma of IPF patients via circRNA microarray analysis.^12^ Zhao group once has selected circANKRD42, circGRHPR, circCDC27, circZMYM2, circARHGAP26 and circTADA2A to prove our data.^3^ Research on these circRNAs will reveal a way of transmitting genetic information mediated by circRNAs and annotate the structure and function of the genome from a perspective different from that of protein‐coding genes in pulmonary fibrosis. Among these circRNAs, the upregulated circRNA‐102,348 was termed as circELP2 because it came from the gene of elongator complex protein 2. Mitochondria are central organelles to numerous biological processes such as cell stress, metabolism, autophagy and cell death, which in turn makes mitochondria have multiple quality control pathways including mitochondrial biogenesis, fusion/division, mitophagy and unfolded protein response processes, to ensure mitochondria to meet the demands of the cell.^13^, ^14^, ^15^ This reason makes mitochondria good targets for the development of therapeutic strategies.^16^, ^17^ However, whether circRNAs regulate pulmonary fibrogenesis through mitochondrial quality control pathway needs further investigation. The present work was designed to research circELP2 biogenesis, function and regulatory mechanism of mitochondrial quality control pathway during pulmonary fibrogenesis.

## 2 MATERIALS AND METHODS

### 2.1 Animal model

The mice treatment in this study was approved by the Animal Ethics Committee of Binzhou Medical University. All the mice were purchased from the Model Animal Research Center of Nanjing University (Nanjing, China). Eight‐week‐old C57BL/6 mice were housed in a specific pathogen‐free animal room and allowed free access to food and water. The fibrotic mouse model was established according to our previous study described.^6^ Briefly, each group contained 10 mice. Pulmonary fibrosis mice were administered with 5 mg/kg bleomycin (BLM) via spraying into the lungs using a Penn‐Century MicroSprayer (PennCentury Inc., Wyndmoor, PA, USA), and the sham group was sprayed with an equal volume of saline in the same manner. On the 28th day following treatment, mice were anaesthetised by intraperitoneal injection of 2.5% avertin. Then, lung samples were collected after perfusion of mice lungs with 4% paraformaldehyde.

### 2.2 Cell culture

The mouse fibroblast L929 cell line and human foetal lung fibroblast MRC‐5 cell line were purchased from American Type Culture Collection (CCL‐171™ and CCL‐1™). The cells were incubated in advanced minimum essential medium containing 10% foetal bovine serum, 1% gluta‐max, 1% NEAA, 1% sodium pyruvate and 100× penicillin/streptomycin, and placed into an incubator containing 5% CO_2_ at 37°C. The cell model was treated with 5 ng/mL transforming growth factor β1 (TGFβ1).

### 2.3 Real time cellular analyses (RTCA)

The cellular proliferation and migration were monitored real‐time using a RTCA DPlus instrument system (ACEA Biosciences, China). Briefly, 5 × 10^4^ cells were incubated in the proliferation plate and migration plate according to the manufacturer's guidelines, respectively. The RTCA instrument automatically recorded the curves of proliferation and migration and calculated the amount of proliferating or migrating cells.

### 2.4 Wound healing assay

Wound healing was analysed by IncuCyte S3 live‐cell analysis system (Essen BioScience, USA). Briefly, 5 × 10^5^ cells were incubated in a 96‐well plate. After overnight culture, the cells were wounded with cell scratcher, washed with PBS, replaced with complete medium culture. Then the cells were placed in the S3 live‐cell analysis system for real‐time dynamic observation. The images were captured by the system software.

### 2.5 RNA fluorescence in situ hybridization (RNA FISH) assay

U6, 18S and circELP2 FISH probes were purchased from Ribo Bio Technology (Guangzhou, China). MRC‐5 cells were fixed with paraformaldehyde on a circular slide in the culture well, punched with 0.5% Triton X‐100. Then pre‐hybridization solution was added after culture at 4°C for 3 min. The denatured probe mix was dropped the culture well and incubated in hybridization oven at 37°C overnight. The hybridization probe solution was aspirated at 42°C. The slides were washed with 2× saline sodium citrate (SSC) and 1× SSC for 3 times, and the cell samples were stained with 150 mL DAPI solution and rinsed with PBS. Finally, a laser confocal microscope was used to record and analyse the fluorescence intensity.

### 2.6 Half‐life analysis for circELP2 and ELP2

After 1 × 10^6^/mL MRC‐5 cells were incubated in cell dishes overnight, the cell culture medium was replaced with 2 mL serum‐free medium. Then, the cell samples were treated with 5 μg/mL actinomycin D for 2, 4, 8 and 12 h. Total RNA was extracted from the collected cell samples for subsequent the quantitative RT‐PCR (qRT‐PCR) analysis according to the manufacturer's guidelines. The forward and reverse primers of circELP2 and ELP2 are as follows: circELP2‐F 5′‐CTGATGAAGAGGAGCTGTTA‐3′ and circELP2‐R 5′‐AGAGAAAGCACTTTCTGAAAAG‐3′; ELP2‐F 5′‐GTCTCTTGTTTGTGCGGCTG‐3′ and ELP2‐R 5′‐CAACCCTTTTCAGGGGGTCA‐3′.

### 2.7 Testing circELP2 from cDNA by agarose gel electrophoresis

Total RNA was extracted by using Trizol reagent. cDNA was synthesised by using the PrimeScriptTM RT kit (TaKaRa, China), then used for PCR to complete cDNA amplification. gDNA was extracted according to the SteadyPure Universal Genomic DNA Extraction kit (Accurate Biology, China). About 0.5 g agarose was added into 50 mL‐TAE solution. The solution was stirred evenly and heated to 100°C. Then the solution was cooled to 50°C and poured into the electrophoresis tank after 2.5 μL nucleic acid dye was added. At room temperature, agarose gel was formed after 30 min. After loading the samples, electrophoresis was carried out for 30 min at a constant voltage 100 V. Images were caught by gel imager (Tanon, China).

### 2.8 RNase R experiment

Total RNA was extracted by using Trizol kit and digested by RNase R (10 U, Geneseed) for 30 min at 37°C. Then cDNA was reversed transcription by PrimeScriptTM RT kit (TaKaRa, China) and amplified by PCR. The PCR production was tested by agarose gel electrophoresis.

### 2.9 RNA antisense purification (RAP) assay

RNA antisense purification experiment was conducted by using RAP kit (Bersinbio, Guangzhou, China). circELP2 probe was synthesised by Bersinbio, and Lac Z probe was as control. The 4 × 10^7^ cells were crosslinked by formaldehyde and glycine and then were collected and lysed with lysate. Probe was added, hybridised for 30 min at 37°C, denatured for 5 min at 50°C and hybridised for 180 min at 37°C. Cells with RNA probe were incubated with magnetic beads for 30 min. Then RNA was eluted, purified and used to detect the expression of miR‐630. circELP2 probe sequence: ATGTCCACAGAGAGAAAGCACTTTCTGAAAAGTTTTTGAACTTCAGGCCACAAAGTATTC; Lac Z probe sequence: GCCTGATGCGGTATTTTCTCCTTACGCATCTGTGCGGTATTTCACACCGCATATGGTGCA.

### 2.10 Chromatin immunoprecipitation (ChIP) assay

Chromatin immunoprecipitation experiment was conducted according to the instruction of simple enzymatic chromatin IP kit (Cell Signalling Technology, USA). The 1 × 10^7^ cells were crosslinked with formaldehyde and glycine and collected. Cell samples were lysed with lysate and sonicated to separate chromatin. YAP/TAZ antibody was added into the cell samples and cultured at 4°C for 12 h. IgG antibody was as the control. The magnetic beads were added into cell samples and cultured at 4°C for 2 h. DNA was eluted and purified. Expression level of mTOR, mLST8 or Raptor were tested by PCR.

### 2.11 MicroCT imaging system for small animal observation

MicroCT imaging system for small animal (PerkinElmer, USA) was turned on for preheating in advance and the parameters were adjusted. Mice were anaesthetised by intraperitoneal injection of 2.5% avertin (0.25 g/kg). After anaesthesia, mice were placed in supine position on the microCT machine. The mouse position was previewed and adjusted to the appropriate position. The images were taken on the microCT imaging system.

### 2.12 mitoROS detection

MitoROS was detected by MitoSOX red reagent (ThermoFisher Scientific, USA). MRC‐5 cells were cultured and treated with TGF‐β1, TGF‐β1 + circELP2 NC, TGF‐β1 + circELP2, over circELP2 NC and over circELP2, respectively. Then the cells were collected, stained with MitoSOX red reagent probe solution for 20 min and detected by flow cytometry (Becton, USA).

### 2.13 Colocalization of LC3 and mito‐mTurquoise2

MRC‐5 cells were seeded in glass bottom cell culture dish. When the cell density reached up to 60%–70%, LC3 reagent and mito‐mTurquoise2 reagent (HANBIO, China) were transfected into cells. After hatching for 6 h, the cells were treated with TGF‐β1, TGF‐β1 + circELP2 NC, TGF‐β1 + circELP2, over circELP2 NC and over circELP2 for 36 h, respectively. The fluorescence images were detected by laser scanning confocal microscope (Zeiss LSM880, Germany). LC3 labelled autophagosome (red) and mito‐mTurquoise2 labelled mitochondria (green).

### 2.14 Statistical analysis

SPSS version 19.0 software was used for statistical analysis. Data were presented as the mean ± SD of at least three independent experiments. Unpaired Student's *t* test was used for experiments comparing two groups, whereas one‐way ANOVA with Student–Newman–Keuls post hoc test was used for experiments comparing three or more groups. *p* < 0.05 was considered as statistical significance.

## 3 RESULTS

### 3.1 circELP2 acted as a pro‐fibrogenic factor to activate fibroblast

**FIGURE 1:**
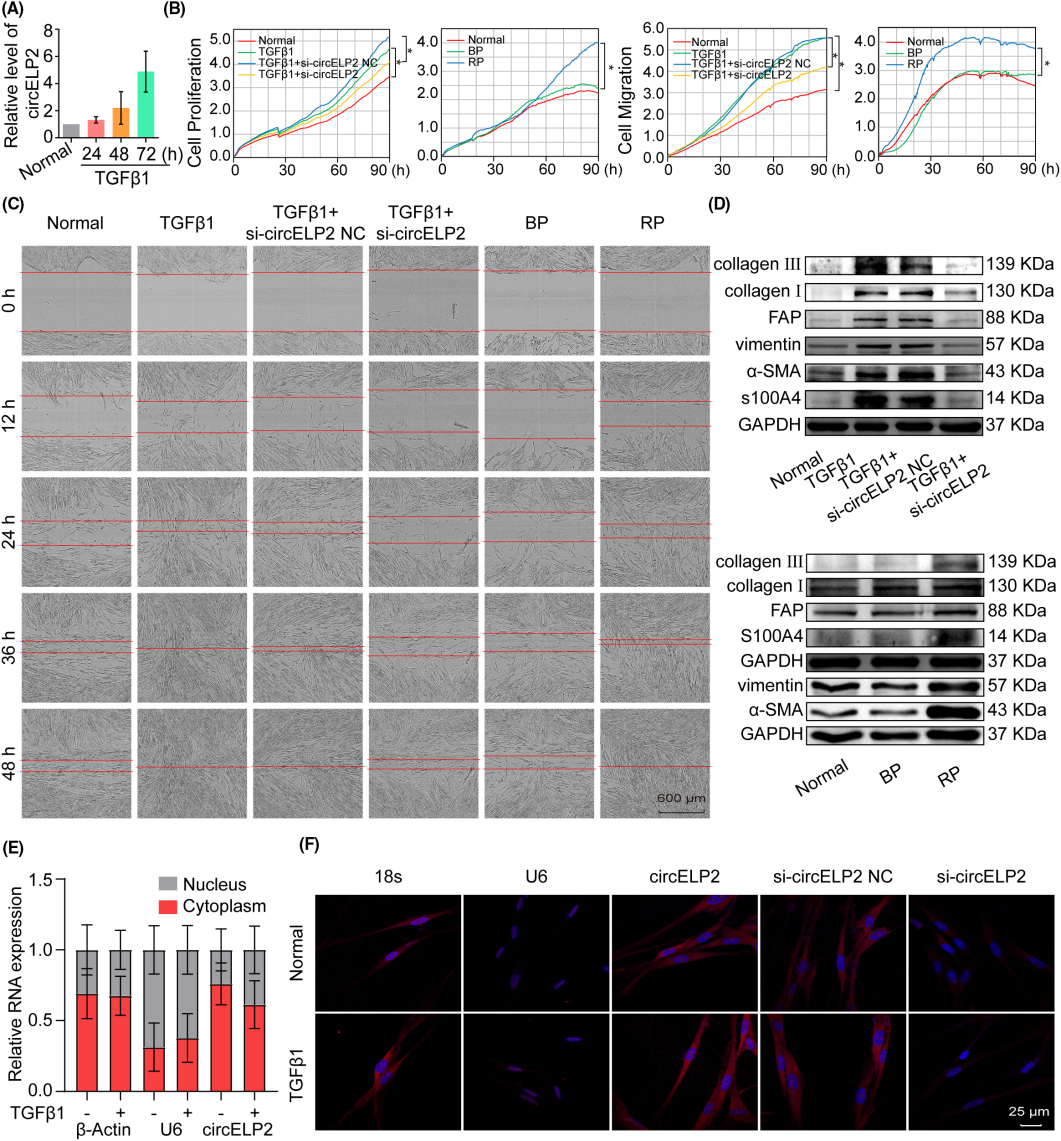
Cytoplasmic circELP2 accelerated fibroblast activation and matrix deposition in TGFβ1‐treated MRC‐5 cells. (A) qRT‐PCR detected that circELP2 increased significantly with time dependence in the TGFβ1‐stimulated group, reaching maximum value in 72 h. (B) The xCELLigence RTCA DPlus instrument monitored that knockdown circELP2 repressed activated fibroblast proliferation and migration. But overexpressed circELP2 enhanced the proliferation and migration. (C) The Incucyte S3 live‐cell analysis system automatically recorded the cell migration and exhibited that the activated fibroblast migration was blocked by circELP2 knockdown and promoted by circELP2 overexpression. (D) Western blot uncovered that the fibrotic and differentiation‐related proteins were inhibited by knockdown circELP2 and promoted by overexpressed circELP2. (E) Nuclear (grey) cytoplasmic (red) separation result manifested circELP2 mainly located in the cytoplasm. β‐Actin served as a cytoplasmic control. U6 served as a nuclear control. (F) RNA FISH images depicted that circELP2 (red) mainly located in the cytoplasm. Silencing circELP2 did not induce circELP2 translocation. 18S and U6 RNA were used as cytoplasmic and nuclear localization markers, respectively. DNA (blue) was stained with DAPI. si‐circELP2 NC indicates si‐circELP2 negative control. BP indicates blank plasmid, and RP indicates the recombinant plasmid of the overexpressed circELP2. Each bar represents mean ± SD (*n* = 6), *p < 0.05.

In our previous work, the differentiated expression of circRNA profiles was tested via circRNA microarray analysis, and highly expressed circELP2 was found in the peripheral blood of IPF patients.^12^ To further explore circELP2, 5 ng/mL TGFβ1 was used to activate MRC‐5. The qRT‐PCR result validated the increase in circELP2 as the action time of TGFβ1 progressed (Figure 1A). On this basis, circELP2 stimulated by TGFβ1 for 72 h in MRC‐5 was recommended for further study.

The abnormal proliferation and migration of activated fibroblast and the matrix deposition were tested by knockdown and knockin circELP2 to evaluate circELP2 function. Findings of the RTCA system showed that the abnormal proliferation and migration were repressed by the circELP2 knockdown but accelerated by the circELP2 knockin (Figure 1B). The scratch wound‐healing assay further confirmed that the abnormal migration was repressed by the circELP2 knockdown but accelerated by the circELP2 overexpression (Figure 1C). In the Western blot results, the circELP2 knockdown reduced the levels of differentiation‐related proteins, such as S100 calcium‐binding protein A4 (S100A4) and fibroblast activation protein (FAP) and matrix fibrotic protein markers, such as α‐SMA, vimentin, collagen I and III (Figure 1D). The above data showed that circELP2 functioned as a pro‐fibrogenic factor to activate fibroblast–myofibroblast differentiation, and caused the abnormal proliferation and migration and matrix deposition.

To better study the regulatory mechanism of circELP2, its cellular location was test. Nucleocytoplasmic separation was performed to demonstrate that the circELP2 was mainly localised in the cytoplasm (Figure 1E). RNA FISH result further confirmed circELP2 cellular location, and the circELP2 knockdown did not cause its translocation (Figure 1F). The data indicated that circELP2 regulation was mainly associated with post‐transcriptional control.

### 3.2 hnRNP L initiated circELP2 reverse splicing biogenesis

**FIGURE 2:**
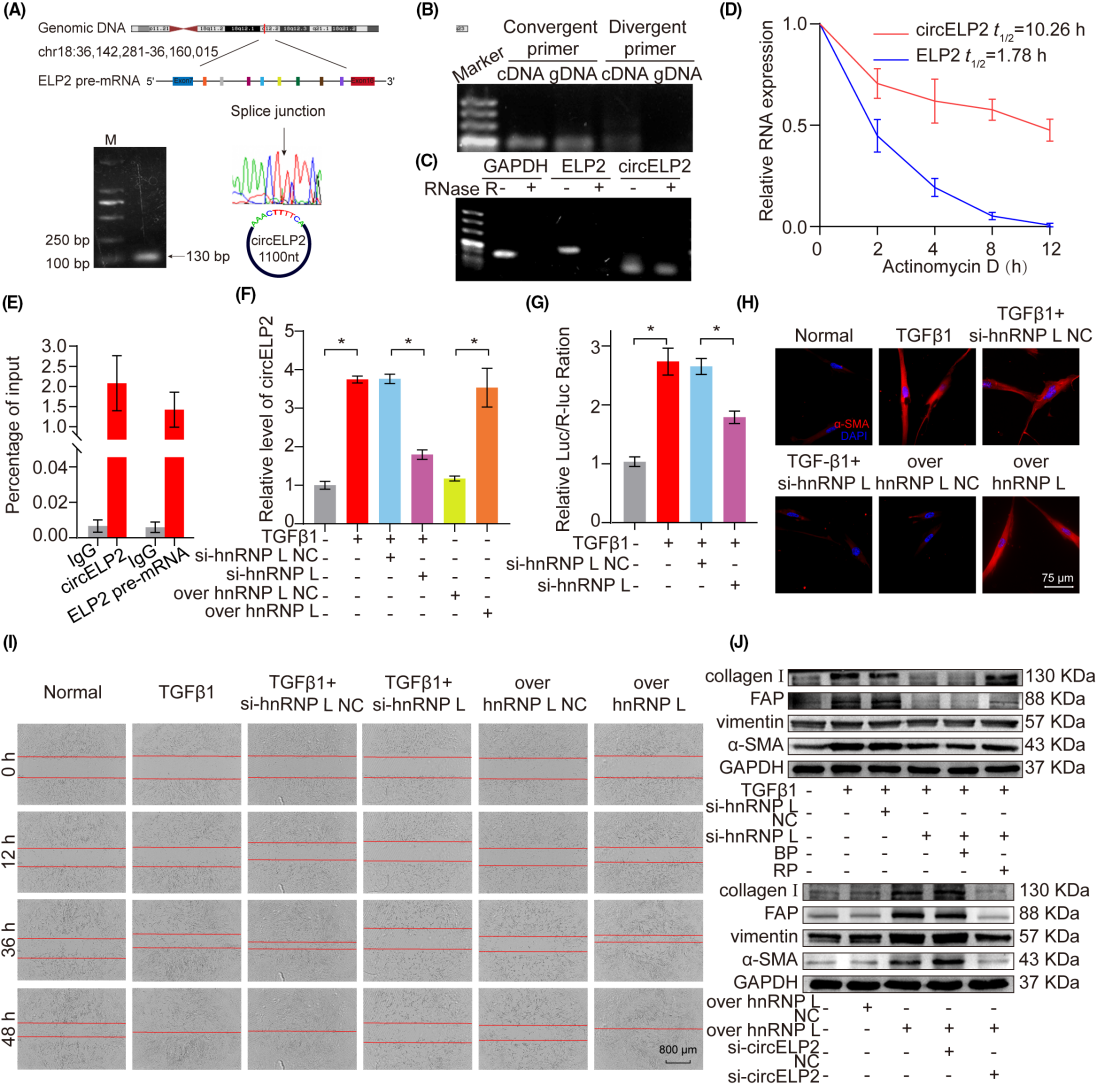
The hnRNP L initiated circELP2 back‐splicing generation. (A) Schematic illustration showing circELP2 produced from its host gene ELP2. Sanger sequencing after PCR with specified primers illustrated the circular junction of circELP2, and its full length was 1100 nt. (B) Agarose gel electrophoresis result revealed that circELP2 generated from cDNA, not gDNA. (C) After RNase R action, circELP2 was detected, but GAPDH and ELP2 were not detected. (D) The actinomycin D experiment indicated that circELP2 was more stable than ELP2. (E) qRT‐PCR analysed that hnRNP L was detectable in the anti‐hnRNP L RIP, but not undetectable in the IgG RIP. (F) si‐hnRNP L inhibited circELP2 and overexpressed hnRNP L promoted circELP2. (G) Dual‐luciferase experiment discovered that si‐hnRNP L repressed circELP2 fluorescence intensity. (H) Immunofluorescence result depicted that silencing hnRNP L made the cellular morphology normal and α‐SMA decreased, but overexpressed hnRNP L worsened the cellular state and α‐SMA increased. (I) The myofibroblast migration was inhibited by si‐hnRNP L and enhanced by overexpressed hnRNP L. (J) circELP2 overexpression reversed the downtrends of fibrotic and differentiation‐related proteins caused by interfering with hnRNP L. si‐circELP2 reversed the uptrends of fibrotic and differentiation‐related proteins induced by hnRNP L overexpression. Each bar represents mean ± SD (*n* = 6), *p < 0.05.

Then, the mechanism of circELP2 upregulation was explored. The circular junction of circELP2 was analysed using PCR products followed by Sanger sequencing. The detected result illustrated that circELP2 was back‐spliced from the seventh to sixteenth exon in the ELP2 pre‐mRNA located in chromosome 18, and the length had 1100 nt (Figure 2A). The agarose gel electrophoresis result showed that the divergent primers amplified the circELP2 from complementary DNA (cDNA), but the genomic DNA (gDNA) had no the target products (Figure 2B). The RNase R and actinomycin D experiments identified that circELP2 was more stable than ELP2 (Figure 2C,D). Heterogeneous nuclear ribonucleoprotein L (hnRNP L), as an alternative RNA splicing factor, is involved in RNA processing.^18^ Thus, MRC‐5 cell lysate and either anti‐hnRNP L or IgG as the IP antibody were analysed by RNA‐binding protein immunoprecipitation (RIP). The purified RNA from RIP was tested via qRT‐PCR. The hnRNP L was observed to be bound with pre‐mELP2 (Figure 2E). Meanwhile, silencing hnRNP L decreased the circELP2 expression, whereas overexpressed hnRNP L increased the circELP2 expression (Figure 2F). The dual‐luciferase experiment validated that silencing hnRNP L repressed the circELP2 fluorescence intensity. These results clarified that circELP2 was binded to hnRNP L (Figure 2G). The immunofluorescence images showed silencing hnRNP L normalised the cellular morphology and decreased the α‐SMA, but overexpressed hnRNP L worsened the cellular state and increased the α‐SMA (Figure 2H). The activated fibroblast migration was inhibited by the silencing hnRNP L but enhanced by the overexpressed hnRNP L (Figure 2I). Then, rescue experiments were performed to prove that the hnRNP L function was dependent on circELP2. The circELP2 overexpression reversed the decreased trends of differentiation‐related and fibrotic protein markers induced by silencing hnRNP L. The knockdown circELP2 reversed the increased trends of these protein markers induced by the knockin hnRNP L (Figure 2J). The findings suggest the ability of hnRNP L to initiate the reverse splicing of circELP2, leading to promote the pro‐fibrogenic effect of circELP2.

### 3.3 circELP2 sponged miR‐630 to promote pulmonary fibrosis

**FIGURE 3:**
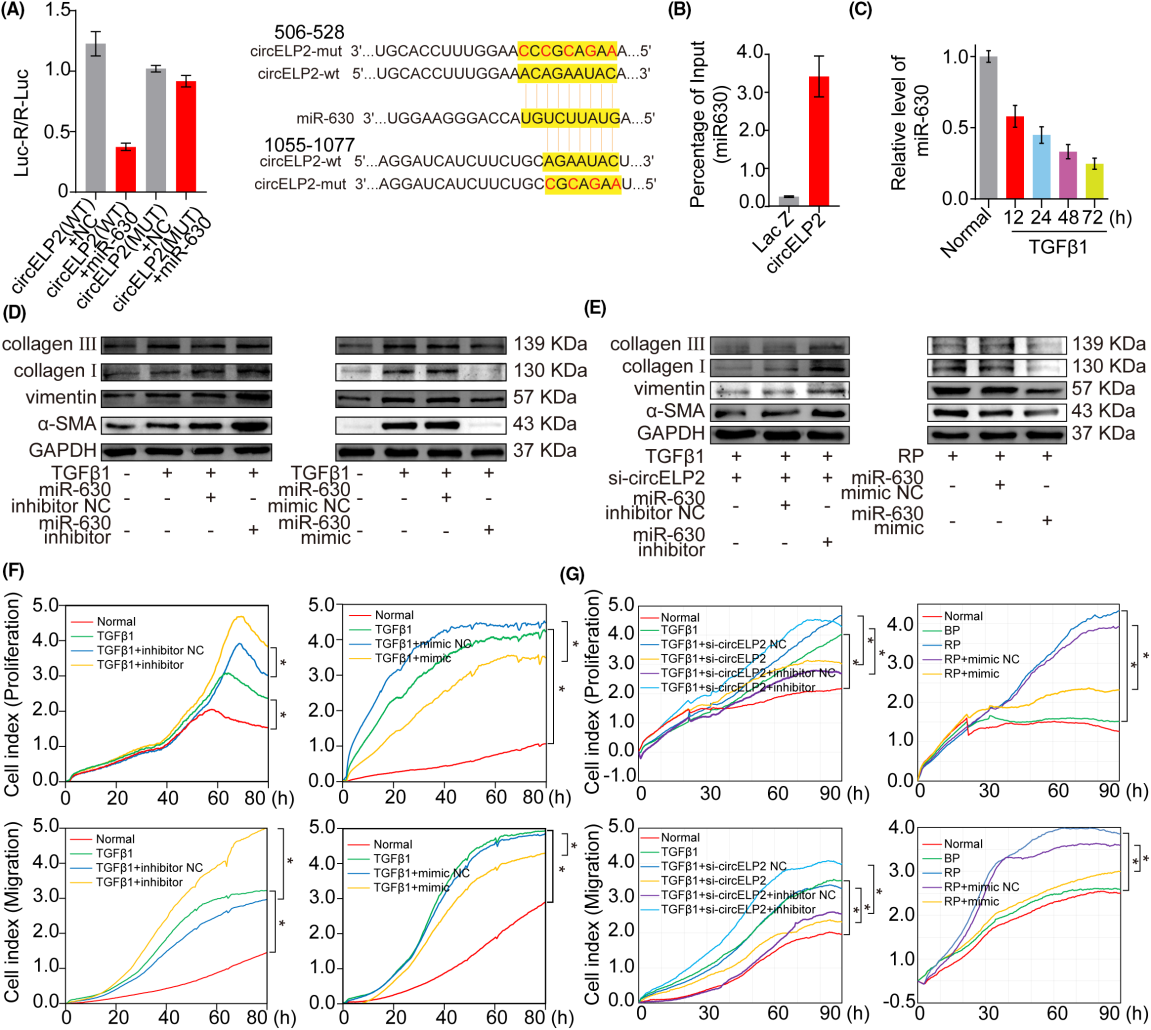
The circELP2 acted as a sponge of miR‐630 to promote pulmonary fibrosis. (A) Firefly and Renilla assay investigated that miR‐630 mimic markedly inhibited the luciferase activity. When the predicted binding site of circELP2 was mutated, miR‐630 mimic did not cause the change of luciferase activity. (B) RAP experiment proved that miR‐630 directly was binded to circELP2. (C) qRT‐PCR detection illustrated that miR‐630 expression was significantly reduced in MRC‐5 cells induced by 5 ng/mL TGFβ1 for 24, 48 and 72 h. (D) miR‐630 inhibitor obviously promoted vimentin, α‐SMA, collagen I and III, and miR‐630 mimic obviously inhibited their expressions. (E) Rescue experiment of Western blot exhibited that miR‐630 inhibitor reversed the downtrend of these fibrotic proteins induced by knockdown circELP2 and miR‐630 mimic reversed the uptrend of these proteins induced by overexpressed circELP2. (F) The proliferation and migration slowed down after miR‐630 mimic transfected into cell samples, but increased after miR‐630 inhibitor transfection. (G) Rescue experiments of proliferation and migration indicated that miR‐630 inhibitor reversed the downtrend of proliferation and migration induced by circELP2 knockdown and miR‐630 mimic reversed the uptrend of proliferation and migration induced by circELP2 overexpression. Each bar represents mean ± SD (*n* = 6), *p < 0.05.

Given that circELP2 is mainly in the cytoplasm, the common regulatory pattern of the circRNA sponging of miRNAs was first explored. After prediction by different types of software (TargetScan, miRanda and miRBase), miR‐630 was found to have a higher binding affinity than the other miRNAs and selected for further study. The dual‐luciferase analysis validated the ability of miR‐630 to bind to circELP2 (Figure 3A). The RAP analysis confirmed the direct combination between miR‐630 and circELP2 (Figure 3B). Then, the miR‐630 function in the pulmonary fibrosis was evaluated. Its expression gradually decreased as the TGFβ1 treatment time progressed (Figure 3C). The Western blot unveiled that miR‐630 mimic prevented the expressions of α‐SMA, vimentin and collagen I, whereas the miR‐630 inhibitor promoted these protein expressions (Figure 3D). The rescue experiment of Western blot elucidated that the miR‐630 inhibitor rescued the downtrend of the fibrotic marker proteins induced by the circELP2 knockdown, whereas the miR‐630 mimic reversed the uptrend of these fibrotic proteins induced by the circELP2 overexpression (Figure 3E). The abnormal proliferation and migration of activated fibroblasts were monitored by a RTCA system, which decreased under miR‐630 mimic treatment but increased under miR‐630 inhibitor treatment (Figure 3F). Then, according to the rescue experiment, the miR‐630 inhibitor rescued the downtrend of the proliferation and migration induced by the circELP2 knockdown, whereas the miR‐630 mimic rescued the uptrend of the proliferation and migration induced by the overexpressed circELP2 (Figure 3G). These findings suggested the dependence of the pro‐fibrotic function of circELP2 on the miR‐630.

### 3.4 circELP2 targeted YAP1 and TAZ depending on miR‐630

**FIGURE 4:**
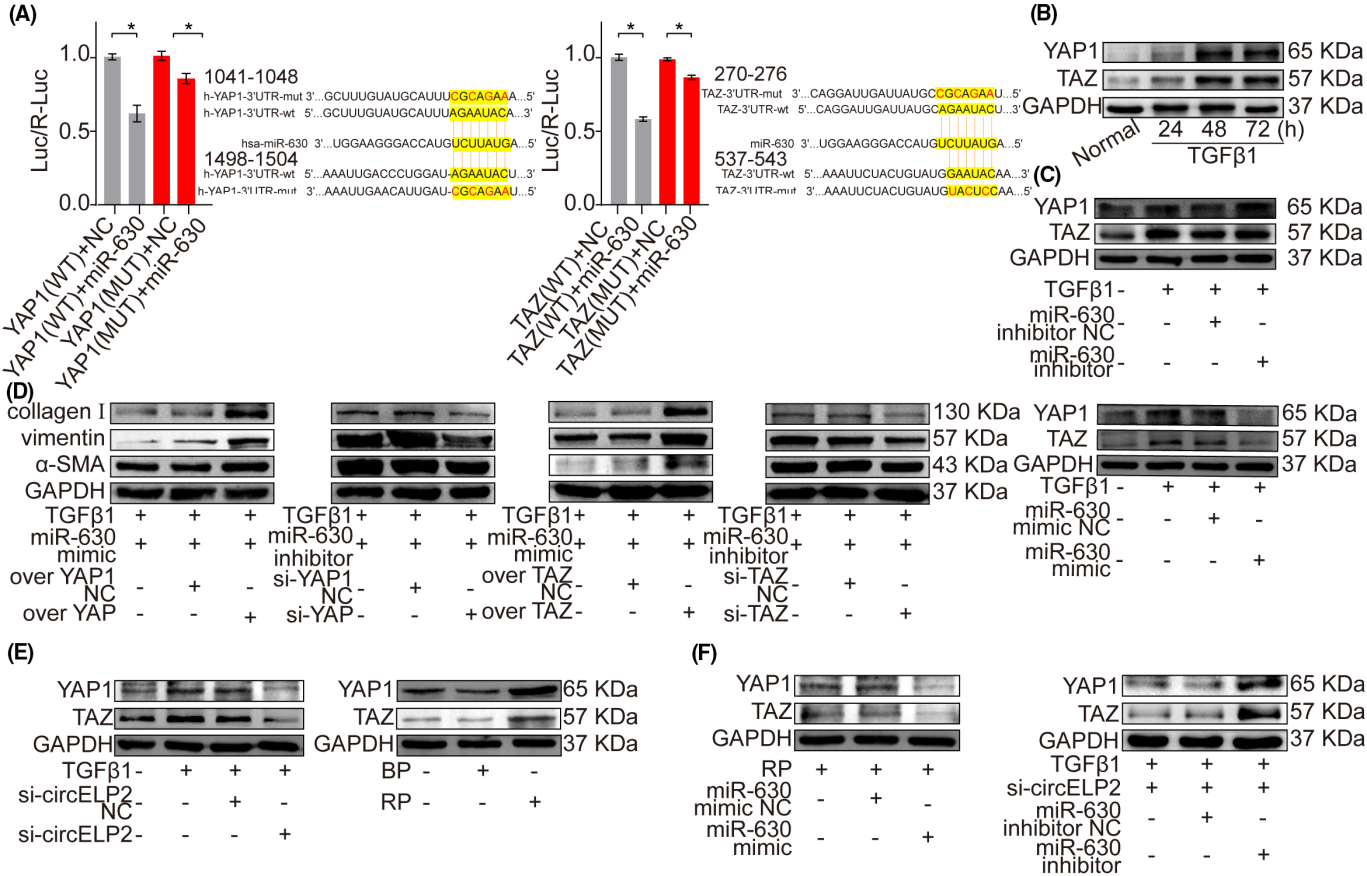
The circELP2 targeted YAP1 and TAZ by sponging of miR‐630 to promote pulmonary fibrosis. (A) Firefly and Renilla dual‐luciferase test indicated that miR‐630 mimic obviously inhibited luciferase activity. When the predicted binding site of YAP1 and TAZ was mutated, miR‐630 mimic did not cause the change of luciferase activity. (B) Western blot elucidated that YAP1 and TAZ obviously increased in MRC‐5 cells induced by 5 ng/mL TGFβ1 at 24, 48 and 72 h. (C) Western blot demonstrated that miR‐630 mimic repressed YAP1 and TAZ expression and miR‐630 inhibitor increased their expression. (D) Rescue experiment presented that overexpressed YAP1 and TAZ rescued the downtrend of the fibrotic proteins induced by miR‐630 mimic. Silencing YAP1 and TAZ rescued the increasing trend of these proteins induced by miR‐630 inhibitor. (E) YAP1 and TAZ were inhibited by knockdown circELP2 and enhanced by highly expressed circELP2. (F) The downward trends of YAP1 and TAZ were reversed by miR‐630 inhibitor and the upward trends of YAP1 and TAZ were reversed by miR‐630 mimic. Each bar represents mean ± SD (*n* = 6), *p < 0.05.

The circRNA sponging of miRNA exerts regulatory function via the target gene. Thus, the miR‐630 target genes were predicted through TargetScan, miRanda and miRBase. According to the analysis, the transcriptional co‐activators Yes‐associated protein 1 (YAP1) and transcriptional co‐activator with PDZ‐binding motif (TAZ) functioned as the target genes. YAP1 and TAZ were further confirmed the miR‐630 target genes via the Firefly and Renilla dual‐luciferase analysis (Figure 4A). The YAP1 and TAZ expression increased as the action time of TGFβ1 progressed (Figure 4B). The miR‐630 mimic repressed YAP1 and TAZ expression, and the miR‐630 inhibitor increased their expression (Figure 4C). The rescue experiment verified the ability of the overexpressed YAP1 and TAZ to reverse the downtrend of the fibrotic marker proteins induced by the miR‐630 mimic. The si‐YAP1 and si‐TAZ rescued the increasing trend of these proteins induced by the miR‐630 inhibitor (Figure 4D). The data indicated that the miR‐630 function depended on the YAP1 and TAZ. Both YAP1 and TAZ were reduced by silencing circELP2 but promoted by highly expressed circELP2 (Figure 4E). By contrast, the downward trends of YAP1 and TAZ caused by the si‐circELP2 were reversed by the miR‐630 inhibitor, whereas the upward trends of YAP1 and TAZ caused by the overexpressed circELP2 were reversed by the miR‐630 mimic (Figure 4F). These trends indicated that circELP2 sponging of miR‐630 targeted YAP1 and TAZ to promote pulmonary fibrosis.

### 3.5 circELP2 targeted mTORC1‐medicated mitochondrial quality control pathway via YAP1/TAZ

**FIGURE 5:**
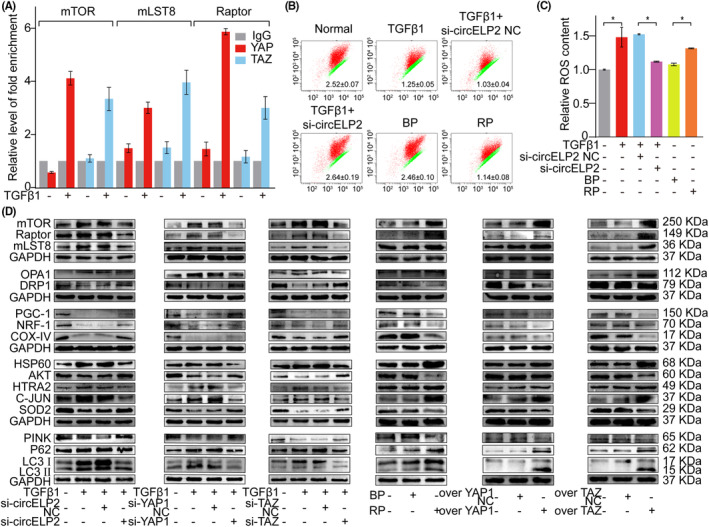
The circELP2 regulated mitochondrial quality control via YAP1/TAZ targeting mTORC1. (A) ChIP result proved that YAP1 and TAZ bound to the promoter regions of mTOR, Raptor and mLST8. (B) Mitochondrial transmembrane potential enhanced under the knockdown circELP2 action, and declined under the overexpressed circELP2 action. (C) ROS declined under the knockdown circELP2 action, and elevated under the overexpressed circELP2 action. (D) circELP2, YAP1 and TAZ activated or inhibited the target genes in mitochondrial quality control pathway such as mitochondrial fusion/fission, mitochondrial biogenesis, mitophagy and unfolded protein response. Each bar represents mean ± SD (*n* = 6), *p < 0.05.

**FIGURE 6:**
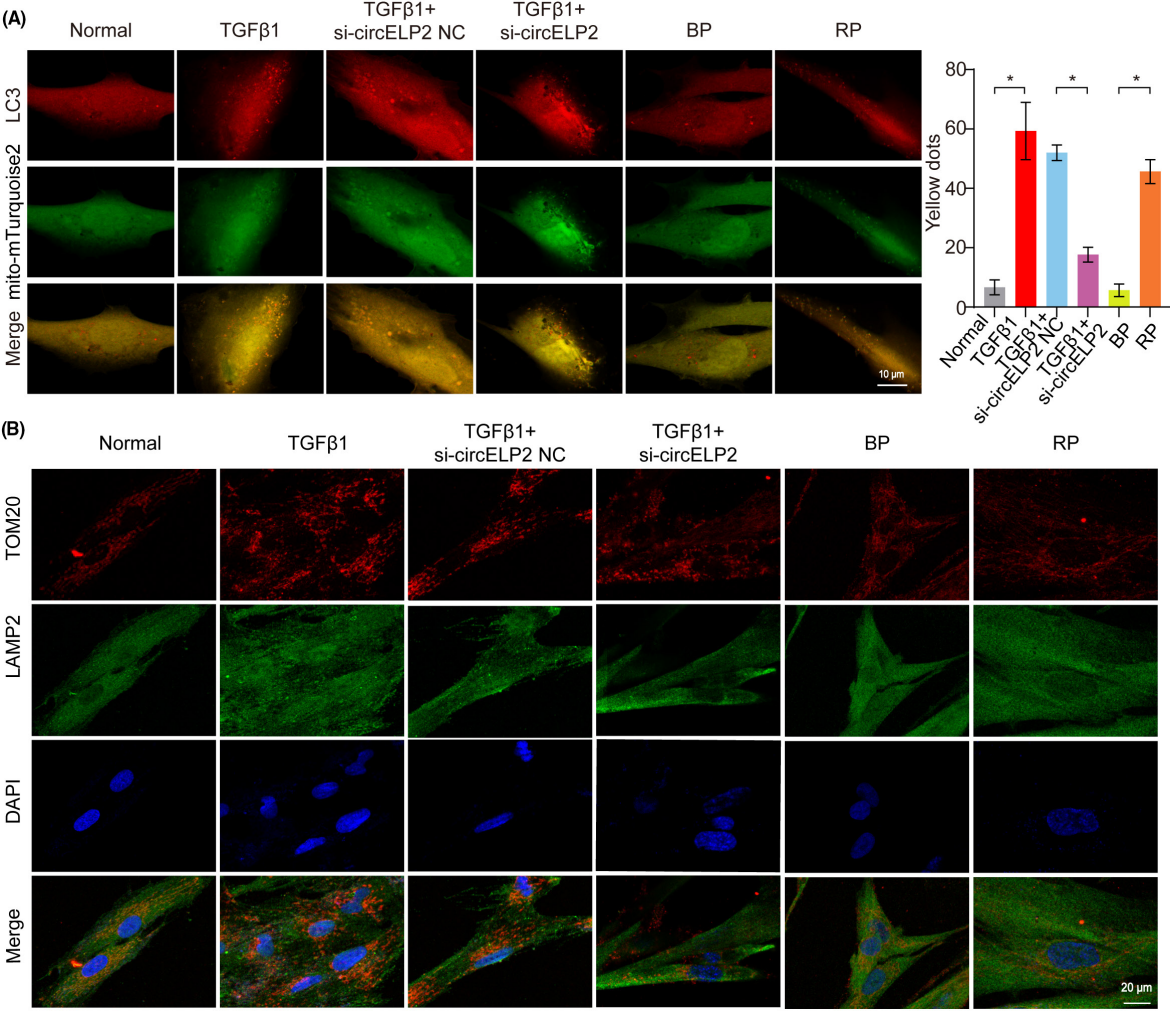
Immunofluorescence colocalization demonstrated knockdown circELP2 enhanced the autophagic flow. (A) Colocalization of mitochondria and autophagosomes reduced by silencing circELP2 and enhanced by overexpressed circELP2. LC3 labelled autophagosome (red); mito‐mTurquoise2 labelled mitochondria (green); the number of yellow fluorescent dots represents the colocalization degree. (B) The colocalization of the mitochondria and lysosome was increased by the knockdown circELP2 but decreased by the overexpressed circELP2. TOM20 labelled mitochondria (red); LAMP2 labelled lysosome (green). Each bar represents mean ± SD (*n* = 3), *p < 0.05.

Yes‐associated protein 1 and TAZ are the trans‐acting factors for gene expression. They can bind to the genes' cis‐acting element to activate or inhibit the target gene. Therefore, the target genes of YAP1 and TAZ were identified via ChIP experiment. The core elements of mammalian target of rapamycin complex 1 (mTORC1), such as mTOR, regulatory associated protein of mTOR (Raptor) and mLST8, were found to be the target genes of YAP1 and TAZ (Figure 5A). mTORC1 participates in the regulation of mitochondrial quality control pathway such as mitochondrial biogenesis, fusion/division, mitophagy and unfolded protein response processes,^19^, ^20^ and mitochondrial dysfunction is widely recognised as a phenomenon of pulmonary fibrogenesis. Therefore, the regulation of circELP2 on mitochondrial quality control via YAP1/TAZ was further explored. The mitochondrial transmembrane potential was increased by the knockdown circELP2, whereas the reactive oxygen species (ROS) were decreased. However, the overexpressed circELP2 promoted ROS but weakened the transmembrane potential (Figure 5B,C). The mTOR, Raptor and mLST8 were decreased by the knockdown circELP2, YAP1 and TAZ, but they were increased by the overexpressed circELP2, YAP1 and TAZ. Dynamin‐related protein 1 (DRP1) and optic atrophy 1 (OPA1) are the proteins of mitochondrial fusion/fission. According to the data, DRP1 was increased by the knockdown circELP2, YAP1 and TAZ and decreased by the overexpressed circELP2, YAP1 and TAZ. OPA1 was decreased by the knockdown circELP2, YAP1 and TAZ and increased by the overexpressed circELP2, YAP1 and TAZ. Peroxisome proliferators‐activated recepter‐coactivatoe‐1 (PGC‐1), nuclear respiratory factor‐1 (NRF‐1) and cytochrome oxidase IV (COX‐IV) are related to mitochondrial biogenesis. The findings showed that PGC‐1, NRF‐1 and COX‐IV were increased by the knockdown circELP2, YAP1 and TAZ, but decreased by the overexpressed circELP2, YAP1 and TAZ. Heat shock protein 60 (HSP60), high temperature requirement protein A2 (HTRA2) and c‐JUN, and AKT and superoxide dismutase 2 (SOD2) are the elements of mitochondrial unfolded protein response. The result showed that AKT and SOD2 were increased by the knockdown circELP2, YAP1 and TAZ, but decreased by the overexpressed circELP2, YAP1 and TAZ. HSP60, HTRA2 and c‐JUN were decreased by the knockdown circELP2, YAP1 and TAZ, but increased by the overexpressed circELP2, YAP1 and TAZ. Autophagy‐related protein PTEN‐induced putative kinase protein I (PINK1), microtubule‐associated protein 1 light chain 3 (LC3) and sequestosome 1 (p62/SQSTM1) are related to mitophagy. PINK was increased by the knockdown circELP2, YAP1 and TAZ, but decreased by the overexpressed circELP2, YAP1 and TAZ. p62, LC3I and LC3II were decreased by the knockdown circELP2, YAP1 and TAZ, but increased by the overexpressed circELP2, YAP1 and TAZ. A combined analysis of the expressions of LC3II/LC3I, p62 and PINK showed the ability of the knockdown circELP2 to enhance autophagic flow (Figure 5D). The immunofluorescence colocalization demonstrated that the colocalization of the mitochondria and autophagosomes was decreased by the knockdown circELP2 but increased by the overexpressed circELP2 (Figure 6A). Combining with our previous studies,^21^ the result suggested that autophagic flow was blocked at autophagosome degradation stage under the treatment of TGFβ1 or overexpressed circELP2, and knockdown circELP2 promoted autophagosome degradation leading to enhanced the autophagic flow. To confirm this point, the colocalization of the mitochondria and lysosome was further explored. The immunofluorescence result showed that the colocalization of the mitochondria and lysosomes was increased by the knockdown circELP2 but decreased by the overexpressed circELP2 (Figure 6B), which further proved that knockdown circELP2 promoted autophagosome degradation. The above findings uncovered that circELP2 targeted mTORC1‐medicated mitochondrial quality control pathway via YAP1/TAZ.

### 3.6 Blocking circELP2‐mediated pathway alleviated pulmonary fibrosis in mice

Finally, whether circELP2‐mediated pathway can be a therapy target of pulmonary fibrosis was evaluated in vivo. Because circELP2 was sifted from IPF patients, the signal pathway of circELP2‐miR‐630‐YAP1/TAZ‐mitochondrial quality control was first confirmed in mouse fibroblast L929 cell line. The data proved that highly expressed humanised circELP2 increased the fibrotic proteins, differentiation‐related proteins and YAP1/TAZ. Proteins in mitochondrial quality control pathway were changed by overexpressed humanised circELP2 and were the same as those in MRC‐5 (Figure S1). Collectively, the effect of the overexpressed humanized circELP2 in L929 cells was consistent with the MRC‐5 results, indicating that the circELP2‐mediated signal pathway is also present in mice. Consequently, the mouse model was used to evaluate the value of therapy target for circELP2‐miR‐630‐YAP1/TAZ‐mitochondrial quality control signal pathway.

**FIGURE 7:**
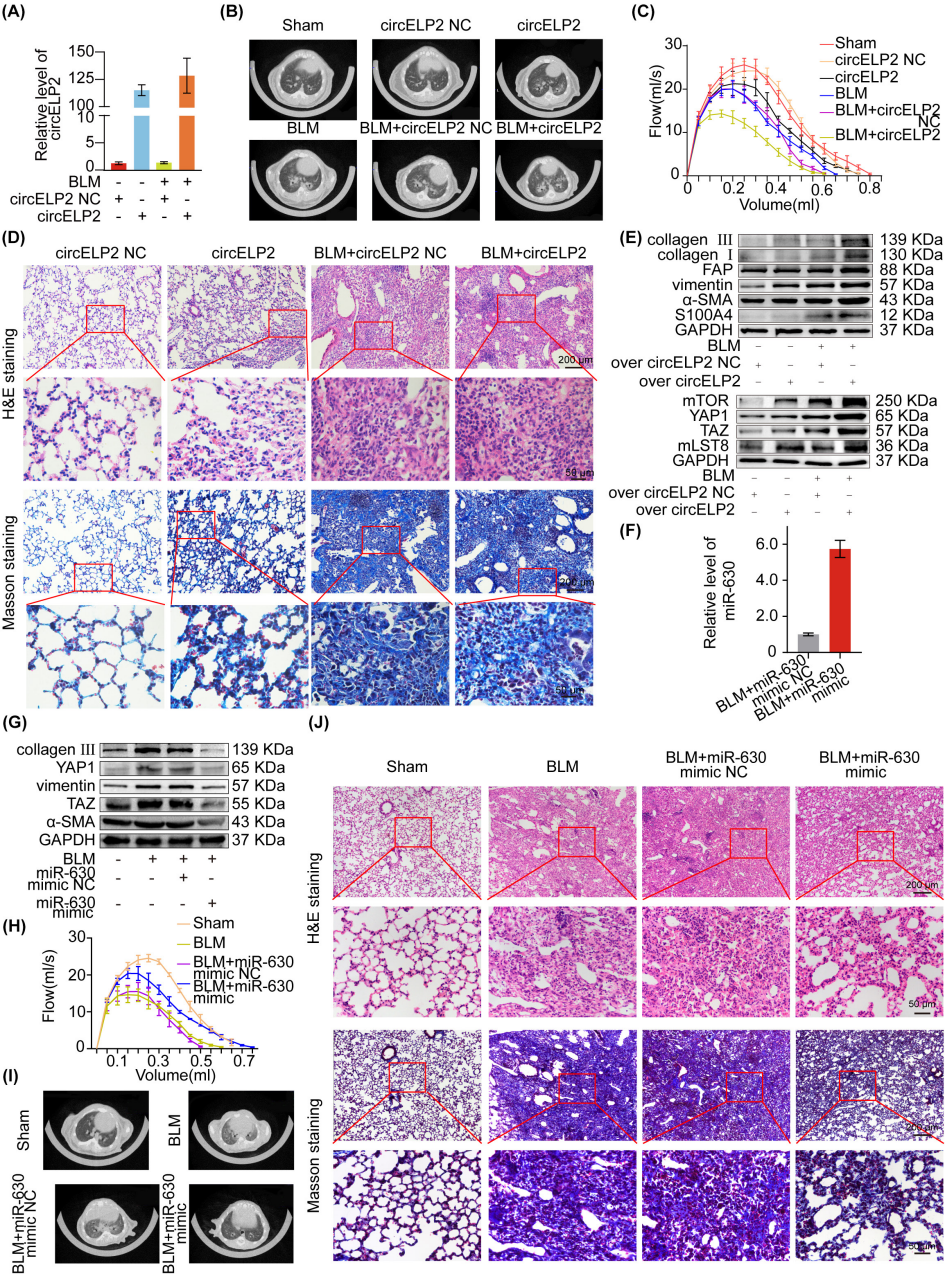
Overexpressed humanized circELP2/ miR‐630 mimic was sprayed into the mouse lung to evaluate circELP2 as a therapeutic target and circELP2‐mediated signal pathway. (A) qRT‐PCR result validated that overexpressed humanized circELP2 increased circELP2 in mice lung. (B) MicroCT imaging system for small animal clarified that the fibrosis degree was evidently higher in highly expressed circELP2 group than control group. (C) Overexpressed circELP2 treatment worsened lung function. (D) Images of H&E and Masson's trichrome staining depicted that the highly expressed circELP2 group had more collagen accumulation, more thickened alveolar walls and more deteriorate fibrotic degree than the control group. (E) Western blot unveiled that the fibrotic proteins were strengthened in highly expressed circELP2 group. The targeting proteins YAP1, TAZ, mTOR and mLST8 also increased in overexpressed circELP2‐treated mice. (F) qRT‐PCR result proved that miR‐630 increased in miR‐630 mimic‐treated mice. (G) The fibrotic proteins and the targeting proteins YAP1 and TAZ decreased in miR‐630 mimic‐treated mice. (H) miR‐630 mimic treatment promoted lung function. (I) MicroCT images demonstrated that the degree of fibrosis in miR‐630 mimic‐treated group was significantly lower than control group. (J) Immunohistochemical images depicted that miR‐630 mimic treatment improved lung structure and reduced collagen deposition.

Given that circELP2 knockdown was not suitable for mice because of the low‐degree homology of humanised circELP2 in mice, the highly expressed circELP2 of adenovirus vectors was sprayed into the mice lung according to a similar study.^22^ The qRT‐PCR result proved the ability of the overexpressed humanized circELP2 to increase circELP2 in mice (Figure 7A). The MicroCT system depicted that the overexpressed circELP2‐treated mice had more patchy opacity and irregular reticular shadows in their lungs (Figure 7B). The overexpressed circELP2 treatment worsened the lung function (Figure 7C). Immunohistochemical staining results depicted that the structure of lung tissue deteriorated and the collagen fibre accumulation increased in the highly expressed circELP2‐treated mice (Figure 7D). The fibrotic and differentiation marker proteins markedly increased in the overexpressed circELP2‐treated mice. The targeting proteins YAP1, TAZ, mTOR and mLST8 increased in overexpressed circELP2‐treated mice (Figure 7E).

In addition, as the role of circELP2 was exerted via miR‐630, the miR‐630 mimic of adenovirus vectors was sprayed into the mice lung. The qRT‐PCR result showed that miR‐630 mimics increased miR‐630 in mice (Figure 7F). The expression of fibrotic proteins and the targeting proteins YAP1 and TAZ decreased in the miR‐630 mimic‐treated mice (Figure 7G). Lung function was improved by miR‐630 mimic (Figure 7H). Morphological observation depicted that the structure of lung was well‐formed and the collagen deposition decreased in the miR‐630 mimic‐treated mice (Figure 7I,J). The data confirmed that blocking circELP2‐medicated pathway alleviated pulmonary fibrosis in vivo, and circELP2 can be a therapy target of pulmonary fibrosis.

## 4 DISCUSSION

Numerous circRNAs are involved in pulmonary fibrogenesis, either as a fibrotic suppressor or a profibrotic factor. But the biogenesis and regulatory mechanism of circRNA in pulmonary fibrosis are still largely unclear. In the present work, we explored the circELP2 biogenesis and its regulatory mechanism on driving pulmonary fibrogenesis. Mechanistic dissection demonstrated that hnRNP L started circELP2 back‐splicing, resulting in a marked increase in circELP2 production. Increasing circELP2 targeted YAP1/TAZ via sponging miR‐630. YAP1/TAZ activated or inhibited genes expression in the mitochondrial quality control pathway by binding to the promoter regions of mTOR, Raptor and mLST8, thereafter accelerating the fibroblast–myofibroblast differentiation and extracellular matrix deposition to promote pulmonary fibrosis.

The circRNAs are produced via the precursor mRNA reverse‐splicing of exon and/or intron of genes. Accumulating evidence indicates that changed circRNA formation participates in various diseases processes.^23^ circRNAs are vary expressed under different physiological and pathological conditions, indicating the participation of different proteins in their generation. Li and colleagues used genome‐wide siRNA screening and an efficient circRNA expression reporter to investigate the proteins contributed to circRNA biogenesis. The nuclear export of NF90/NF110 to the cytoplasm upon viral infection is found to lead reduces the circRNA formation.^24^ According to the stratified eCLIP hits across the genome with circRNA loci coordinates, the splicing factor proline/glutamine‐rich (SFPQ) and non‐POU domain‐containing octamer‐binding (NONO) proteins are specifically enriched in the flanking introns of distal‐Alu‐long‐intron (DALI) circRNAs. Furthermore, in the RIP, SFPQ and NONO promoted a biogenesis of DALI circRNAs, such as circCDYL, circARHGAP5 and circEYA1.^25^ The transacting RNA‐binding factor quaking (QKI) activated the circNOTCH1 generation. Increasing the circNOTCH1 promotes G protein‐coupled oestrogen receptor‐induced tumour growth through YAP1/TEAD signalling in non‐small‐cell lung cancer.^26^ The hnRNP family, as one of the RNA alternative splicing factors, regulates circRNA biogenesis through reverse splicing. Previously, Fei and colleagues defined the hnRNP L‐bound RNA atlas via RIP coupled with next‐generation sequencing and identified hnRNP L as a prostate cancer dependency that could regulate RNA alternative splicing.^18^ The RNA‐ and eCLIP‐sequencing revealed that the function of hnRNP M is preferentially bind to GU‐rich elements in the long‐flanking proximal introns of its target homeostatic genes, control the circRNA generation and splice the fidelity to sustain cancer cell fitness.^27^ The crosstalk of circPCNX with AU‐rich element RNA‐binding factor 1 (AUF1), also known as hnRNP D, can selectively prevent the AUF1 binding to p21 mRNA, which promote the p21 mRNA stability and translation into protein. Therefore, ultimately, the cell growth is inhibited.^28^ However, the knowledge about RNA‐binding proteins and their related circRNA processing steps remains elusive, especially in pulmonary fibrosis. Our study has proven that hnRNP L can activate circELP2 biogenesis by promoting pre‐mELP2 reverse splicing.

The regulatory mechanisms of circRNAs are mostly dependent on their cellular localization. Cytoplasmic circRNAs play a critical regulator in the processes of gene expression, such as the sequestration of proteins or miRNAs, mRNA stability and translation efficiency. Cytoplasmic circVGLL3, originating from the third exon of the vgll3 gene, markedly enhances the osteogenic differentiation of adipose‐derived mesenchymal stem cells via the circRNA‐vgll3/miR‐326‐5p/Itga5 signal pathway.^29^ The other cytoplasmic circIGHG can be generated from an overlap region between the genes of IGHG1 and IGHG3 (IGHG3 exons 2 to 9 and IGHG1 exons 1 and 2) on the minus‐strand of chromosome 14 in human genome, whose spliced sequence length is 27,944 bp. High circIGHG promotes epithelial–mesenchymal transition through sponging miR‐142‐5p to control IGF2BP3, thereafter increasing the aggressiveness of oral squamous cell carcinoma.^30^ Our study demonstrated that cytoplasmic circELP2‐mediated mitochondrial quality control pathway accelerated pulmonary fibrosis via targeting YAP1/TAZ by sponging miR‐630.

Yes‐associated protein 1 and TAZ are the downstream effectors of Hippo pathway, which facilitate gene transcription to generate biological effects by translocating to the nucleus. The Hippo‐YAP1/TAZ axis can control organ size and tissue homeostasis through regulating cell proliferation, differentiation and apoptosis.^31^, ^32^ Therefore, YAP1/TAZ has been a promising target for preventing abnormal remodelling in numerous diseases.^33^, ^34^, ^35^ Guérin A et al. once have reported that limb expression 1 (LIX1) downregulation alters mitochondrial quality control pathway to control the fate of digestive mesenchyme‐derived cells by regulating YAP1 and TAZ activity.^36^ Here, we presented that YAP1 and TAZ can bind to mTORC1 to activate or inhibit the target genes in mitochondrial quality control pathway. Mitochondrial quality control plays a crucial role not only in modulating cellular activities and homeostasis but also ensuring mitochondrial proteome, such as high plasticity that let the adjustment of mitochondrial function to cellular and molecular requirements.^37^ Failure in ensuring the quality control of components causes changes in many signalling pathways and cell phenotypes, such as pulmonary fibrotic pathway and altered stem cell function, which jointly drive the pro‐fibrotic genes expression leading to lung fibrogenesis.^38^ Previously, Zhao et al. found that the mitochondrial‐located circRNA SCAR inhibited mitochondrial ROS production and fibroblast activation in patients with nonalcoholic steatohepatitis. Mechanistically, lipid overload inhibits PGC‐1a via the endoplasmic reticulum stress‐induced C/EBP‐homologous protein. circRNA SCAR, when regulated by PGC‐1a, binds to ATP5B and closes the mitochondrial permeability transition pore (mPTP) through preventing CypD–mPTP interplay.^39^ However, the process of how mitochondrial quality control regulates pulmonary fibrogenesis under the action of circRNA is largely unknown. The mechanism of circELP2 promoted pulmonary fibrosis via mitochondrial quality control can enrich the understanding of mitochondrion‐related circRNA and pulmonary fibrosis.

## 5 CONCLUSIONS

To sum up, this study unveiled that circELP2 biogenesis and function as a pro‐fibrogenic factor to accelerate fibroblast–myofibroblast differentiation and extracellular matrix deposition by targeting miR‐630‐YAP1/TAZ‐mitochondrial quality control pathway. Blocking circELP2‐mediated signal pathway can alleviate pulmonary fibrogenesis and circELP2 can be a therapy target for lung fibrosis treatment.

## AUTHOR CONTRIBUTIONS

**Songzi Zhang:** Methodology (equal); project administration (equal). **Diwei Tu:** Data curation (equal); formal analysis (equal). **Weili Liu:** Data curation (equal); formal analysis (equal). **Ruiqiong Li:** Data curation (supporting); formal analysis (equal). **Mengqi Jiang:** Writing – original draft (equal). **Xinglong Yuan:** Writing – original draft (equal). **Jianlin Luan:** Writing – original draft (equal). **Hongbo Li:** Methodology (equal); project administration (equal). **Xiaodong Song:** Methodology (equal); project administration (equal). **Changjun Lv:** Methodology (equal); project administration (equal).

## FUNDING INFORMATION

This work was supported by the National Natural Science Foundation of China (82370094, 82370079, 82170085, 81970064 and 81870001), the Natural Science Foundation of Shandong Province (ZR2020MH009 and ZR2020MH010).

## CONFLICT OF INTEREST STATEMENT

The authors declared no potential conflicts of interest with respect to the research, authorship and/or publication of this article.

## Supporting information


Figure S1.

## Data Availability

All data generated or analysed during this study are included in this published article and its supplementary information files.
