# Supplementary figures and images for: circELP2 reverse‐splicing biogenesis and function as a pro‐fibrogenic factor by targeting mitochondrial quality control pathway
Source: J Cell Mol Med. 2023 Dec 30;28(3):e18098. doi: 10.1111/jcmm.18098 (PMC10844706; doi:10.1111/jcmm.18098)

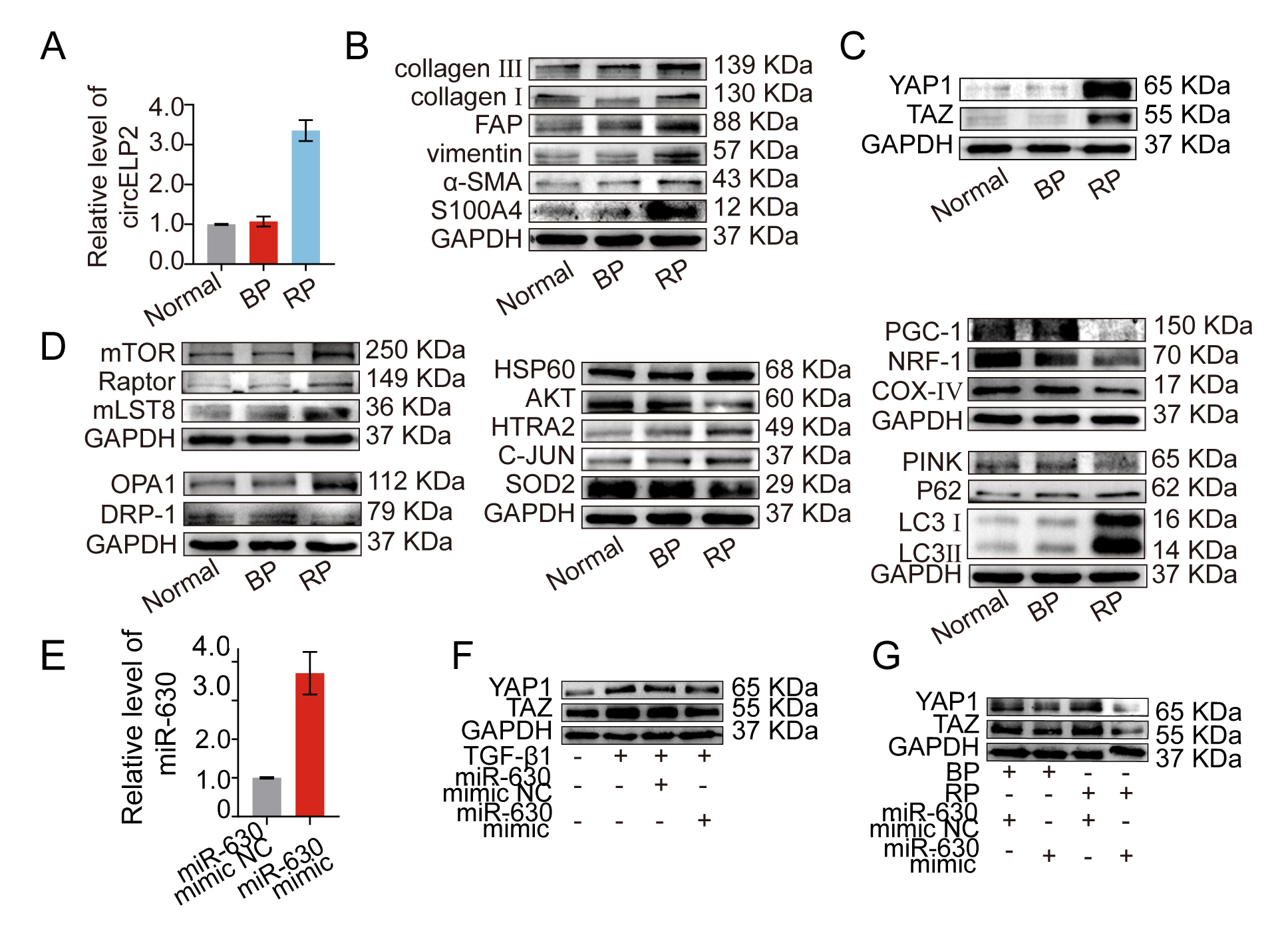

Supplement: Supplementary file 1 — Figure S1. [file JCMM-28-e18098-s001.tif]
